# Recent trends in vision impairment certifications in England and Wales

**DOI:** 10.1038/s41433-020-0864-6

**Published:** 2020-04-14

**Authors:** Farzana Rahman, Antra Zekite, Catey Bunce, Hari Jayaram, Declan Flanagan

**Affiliations:** 1 Moorfields Eye Hospital NHS Foundation Trust, London, UK; 2 NIHR Moorfields Biomedical Research Centre, London, UK; 3 King’s College London, London, UK

**Keywords:** Vision disorders, Epidemiology

## Abstract

**Background:**

The Certificate of Visual Impairment (CVI) provides essential data for preventable sight loss indicators as part of the Public Health Outcomes Framework (PHOF) published annually by the Department of Health. Trends in CVI certification rates can provide information on the effectiveness of current services and treatments and may be used to guide allocation of resources, and is the only such indicator within ophthalmology. This study aimed to compare recent trends in new vision impairment certifications in 2017/18 against prior baseline data in England and document trends in new certifications in Wales.

**Methods:**

PHOF data from 2010/11 and 2017/18 were examined with respect to preventable sight loss indicators: age-related macular degeneration (AMD) (Indicator E12a), glaucoma (Indicator E12b), diabetic eye disease (Indicator E12c) as well as the total numbers of certifications (Indicator E12d).

**Results:**

In 2017/18, the rate of new CVI certifications was 41 per 100,000 population which has reduced from 43/100,000 in 2010/11 in England. Certifications for AMD reduced from 132/100,000 in 2010/11 to 107/100,000 in 2017–18. Certifications for glaucoma have remained stable at 13/100,000 in 2017/8. Certifications for diabetic eye disease have declined from 4/100,000 in 2010/11 to 3/ 100,000 in 2017/18. The number of vision impaired individuals that each Clinical Commissioning Group (CCG) has to support varies from 8 to 82 per 100,000 population.

**Conclusions:**

There has been a significant decrease in the rate of all CVI certifications particularly from AMD and diabetic retinopathy. However, maintaining this will require changes in the way care is delivered as the elderly population, which is at greatest risk of preventable sight loss, is projected to increase by 50% over the next 20 years. Inherited retinal diseases are now the leading cause of sight loss in the working age population. CVI data demonstrate the need for CCGs to tailor their investment in ophthalmic services to the needs of their specific patient populations. It is important that all ophthalmologists continue to provide accurate CVI data in order to help support the future equitable allocation of adequate resources to reduce avoidable vision loss.

## Introduction

The certification of sight loss is an important means of identifying patients in need of support and provides an indicator of vision impairment both in local communities and at a national level. There has been a record of patients with sight loss in England kept since 1851 [1, 2], with the declaration of blindness on census returns up until 1911. The Blind Person’s Act in 1920 initiated a formal register of patients with sight loss and introduced certain benefits, with any medical practitioner then able to register a patient. The BD8 form was introduced in the 1930s which then specifically required any ophthalmologist to provide certification, with the consultant ophthalmologist designated as being responsible from 1955. The National Institute for Health and Care Excellence (NICE) Quality standard [QS180], Serious eye disorders, Quality statement 6: *“Certificate of Vision Impairment”* approved in February 2019, requires that adults with serious eye disorders are given a Certificate of Vision Impairment (CVI) as soon as they become eligible [3].

The original BD8 form facilitated official documentation of sight impairment. However, due to concerns over delayed access to services, the BD8 form was changed to the CVI. In Wales this is known at the CVIW and similar systems are in place in both Scotland and Northern Ireland. The CVI process provides a pathway through which an individual with vision impairment can access certain services through their local authority without being fully registered, although registration might provide additional benefits. The CVI replaced the term ‘blind’ with ‘severely sight impaired’. A consultant ophthalmologist can certify a patient as ‘sight impaired’ or ‘severely sight impaired’ and the process encourages patients with sight impairment to live as independently as possible. With patient consent, the CVI data are then passed to the patient’s general practitioner and local council for registration and to the Royal College of Ophthalmologists Certifications Office based at Moorfields Eye Hospital for epidemiological analysis.

Up-to-date information on recent trends in CVI certifications provides important information on visual impairment in local communities as well as information on the impact of advances in treatment both locally and nationally.

Certification of vision impairment is dependent on visual acuity and visual field criteria. ‘Severe Sight Impairment’ (previously labelled ‘blind’) is legally defined by the National Assistance Act 1948 as “a person so blind as to be unable to perform any work for which eyesight is essential”. The Department of Health and Social Care identifies the following criteria for certifiable severe sight impairment:

- Visual acuity < 3/60 Snellen with a full visual field;
- Visual acuity 3/60–6/60 Snellen with severe visual field loss;
- Visual acuity > 6/60 Snellen with very reduced field, particularly in the inferior visual field.

‘Sight Impairment’ is not legally defined but classed as:

- Visual acuity 3/60–6/60 Snellen with full field of vision;
- Visual acuity 6/60–6/24 Snellen with moderate visual field loss or central blurring of vision;
- Visual acuity > 6/18 Snellen with a large area of visual field loss.

### The Public Health Outcomes Framework in England

The Public Health Outcomes Framework (PHOF) ‘sets out a vision for public health that is to improve and protect the nation’s health and improve the health of the poorest fastest’, essentially laying out the government’s priorities for healthcare. This was first published by the Department of Health in 2012, with a framework of health indicators to understand how the nation’s health is being improved as well as highlighting any inequalities. New baseline sight loss indicator data from 2010/11 to 2011/12 was published in August 2013 [4]. The PHOF aims to improve the nation’s public health through increasing healthy life expectancy and reducing differences between communities.

### CVI certifications and the PHOF in England

‘Preventable sight loss’ is a key indicator within the domain of ‘healthcare and premature mortality’. The CVI provides essential data and epidemiological information for the preventable sight loss indicator which aims to reduce the number of people with this outcome. Within ophthalmology this is the only such indicator and is essential for our understanding of current trends in vision impairment. It is used to inform policy and can also be used to support the case for specific investment or disinvestment.

This study aimed to compare recent trends in new vision impairment certifications in 2017/18 against prior baseline data from England and also document trends in new certifications in Wales using new data released by the Welsh Government.

## Methods

Data published by Public Health England on the PHOF and CVI data published by the Welsh Government were analysed. The following preventable sight loss indicators were examined: age-related macular degeneration (AMD) (Indicator E12a), Glaucoma (Indicator E12b), Diabetic Eye Disease (Indicator E12c) as well as total numbers of certifications (Indicator E12d). The most recent data published for the preventable sight loss indicators covers the period 2017/18. Data for this period were compared with figures through the years from 2010/11 onwards.

Information taken from the PHOF is not true prevalence data because it is not population but hospital based, however is representative of the incidence of new cases of certifiable vision impairment. In order for a patient to become certified, access to healthcare services is necessary in order be seen by a consultant ophthalmologist. The true population prevalence of sight loss, including those in long term care or in institutions where sight loss has not been identified, can only be accurately determined through a comprehensive population-based screening programme.

## Results

There were 22,844 new certifications in the year 2017/18 in England, equivalent to 439 new vision impairment certifications every week. In 2017/18, the rate of new certifications was 41 per 100,000 population, which has reduced in comparison with 43 per 100,000 population in 2010/11.

The certification rate for AMD has reduced from 132 per 100,000 in those aged 65 or over in 2010/11, to 107 per 100,000 aged 65 or over in the year 2017–18. Certifications for glaucoma, remain unchanged at 13 per 100,000 for patients aged 40 or over in 2017/8, with no statistically significant change since 2010/11. Certifications for diabetic eye disease have declined from 4 per 100,000 aged 12 or over in 2010/11, to 3 per 100,000 aged 12 or over in 2017/18.

## Discussion

### CVI certification rates

The CVI form was updated and simplified in 2017 to reduce difficulties and delays in the certification process. With improved patient education, patients are now more aware of certification and its benefits. The reduction in the rate of new CVI certifications is undoubtedly, at least in part, due to better access to treatment, earlier diagnosis and new effective treatments for conditions causing preventable sight loss. However, it is important to be aware of other factors that can influence CVI certification rates. Heavy workloads in busy clinics may prevent discussion of the benefits of certification with patients. In addition, not all clinicians are aware of these benefits and therefore may not discuss them with patients and their relatives. Such factors are difficult to measure and are very variable, making accurate interpretation of CVI data challenging.

### AMD certifications

With an ageing population, the demand for AMD care is rapidly increasing and will continue to do so. Increasing awareness of AMD by patients and optometrists, combined with integrated pathways of care bridging community care with Hospital Eye Services using electronic referral systems, are ensuring that many patients are seen and treated promptly leading to improved clinical outcomes. Multidisciplinary teams of ophthalmologists, optometrists, nurse injectors and technicians now provide safe, effective high quality care to large numbers of patients. High resolution imaging allows more accurate diagnosis and better information on responses to treatment which leads to improvements in treatment strategies and ultimately better outcomes.

### Glaucoma certifications

Diagnosis of patients with glaucoma is often late in the disease course due to the asymptomatic nature of the condition. Improved patient education and awareness is essential to ensure that patients at increased risk of glaucoma [5, 6] such as those with a family history of the condition and those of African or Asian heritage have annual community based screening.

Risk stratification is essential to ensure that patients at a high risk of progression are prioritised with failsafe mechanisms to ensure that they are always reviewed at appropriate intervals and are not “lost to follow up”. The recent report from the Health Service Investigation Branch demonstrates that review arrangements are still inadequate in many glaucoma services in England [7].

Commissioners and providers of ophthalmic care need to develop closer collaborations in order to develop robust pathways that can bridge community and hospital care. This will ensure that patients at a lower risk of progression are reviewed by suitably qualified professionals at appropriate intervals.

### Diabetic eye disease certifications

The following factors have contributed to the observed reduction in certifications attributed to diabetic eye disease:

- The NHS Diabetic Eye Screening Programme (DESP) was established across the UK in 2008 and is an example of a highly effective service which has ensured that most diabetic patients in the UK with ocular complications are detected and treated in a timely manner. This has made a major contribution to the reduction in sight loss secondary to diabetic retinopathy [8]. When the DESP was introduced, diabetic eye disease was the leading cause of sight loss in the working age population, but this is no longer the case with inherited eye disease now the commonest cause in this group [9].
- The widespread availability of new effective treatments for diabetic macular oedema.
- The development of multidisciplinary teams to deliver intravitreal therapies.
- The Quality and Outcomes Framework, introduced in 2004 [10, 11], led to a marked improvement in the overall management of diabetes by incentivising and resourcing general practices to meet key performance indicators.

Advances in imaging technology, particularly ultra-widefield fundus imaging and optical coherence tomography combined with technology to allow rapid transfer of large image files, will facilitate more accessible screening at multiple sites and ultimately automated analysis of images [12]. Increasing the screening interval to 2 years in low risk groups will allow resources to be directed towards groups at higher risk of sight loss. Particular attention should be paid to identifying individuals who have difficulty accessing screening services, have multiple comorbidities or are at risk of being “lost to follow up” due to relocation as these groups often have poor outcomes.

### Beyond the PHOF

**Fig. 1:**
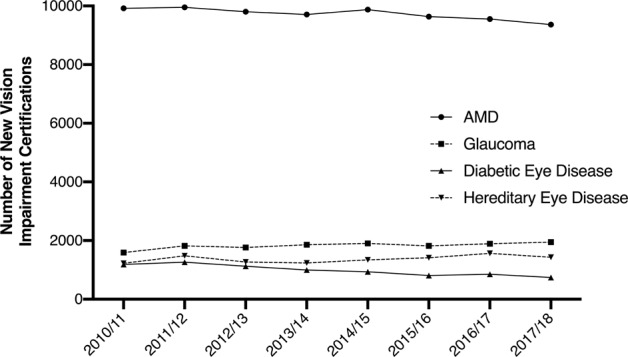
Summary of trends in vision impairment certifications in England by single main cause from 2010 to 2018. The data provided by the Royal College of Ophthalmologists, c/o Certifications Office, based at Moorfields Eye Hospital, captured by the Certificate of Vision impairment (CVI) are Department of Health and Social Care copyright and this work was made possible by collaboration with the Royal College of Ophthalmologists. Any views expressed in this publication/document are those of the author(s) alone and not necessarily those of the Department of Health and Social Care.

CVI data from England and the Welsh Government, managed by the Royal College of Ophthalmologists, allow more detailed analysis of the causes of sight loss than what is currently in the PHOF. Trends in vision impairment certifications over the past 10 years show that inherited retinal diseases are now the leading cause of sight loss in the working age population (Fig. 1), which is in keeping with published data [9]. This is of huge interest to researchers who are now developing treatments for these diseases. This pattern also shows an increase over time which demonstrates the need for ongoing investment in research into inherited retinal disorders. The reason for this increasing trend is multifactorial. It could potentially be explained by greater awareness of such conditions, prompting increased presentation to hospital eye services, along with improved certification overall as already discussed. Consanguinity is also likely to be a contributor to this trend [9].

One in ten cases of vision impairment in the UK are thought to be secondary to inflammatory eye disease [13]. Accurate information on visual impairment from this condition is particularly important as treatment that is safe, clinically and cost effective is now available and many of the patients are young.

### CVI rates in children

Certification in children is of particular importance as the practical support offered as a result can help support development in motor, language, cognitive and social skills, with early intervention being essential for better outcomes [14]. Children who have a CVI completed are not necessarily registered with their local authority. CVI rates in children are not published on PHOF. Information of paediatric CVIs can however be provided by the Certifications Office. Certification of children has increased. In 1999/2000, the estimated incidence of certification was 8.2 (95% CI: 7.7–8.8) per 100,000 children. In 2007/2008, the estimated incidence was significantly higher at 10.1 (95% CI: 9.5–10.7). Since then a trend of increasing incidence has been observed until 2014/2015 when the estimated incidence was 13.3 (12.6–14.0). Hereditary retinal dystrophies, cerebral visual impairment and nystagmus were the most common causes of certifiable sight impairment in children in 2014/2015 [15].

Accurate certification rates in the paediatric population may be more challenging as certification may often be delayed if there is a possibility of vision improvement as the child gets older. The process may also be more difficult and take longer in cases of developmental delay. Aside from hereditary eye disease, survival rates of premature babies have improved and will continue to do so due to advances in medical care. As treatments for retinopathy of prematurity and cerebral visual impairment improve, sight impairment rates may also improve but medical advances will continue and so the relationship with CVI rates will remain dynamic.

### Geographical variations

There are geographical variations in the rates of the preventable sight loss indicators. These may be due to real differences in the incidence and prevalence of sight impairment, differences in how local authorities support individuals with visual impairment, variable certification rates by ophthalmologists and variations in provision of Eye Clinic Liaison Officers (ECLOs) support to eye clinics.

**Fig. 2:**
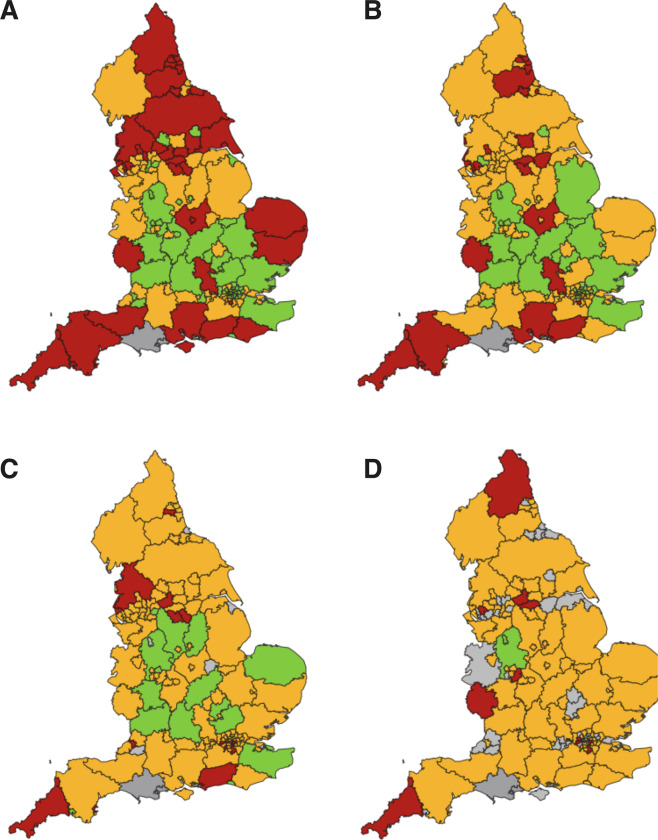
Preventable sight loss certifications in England (2017/18)—Geographical Distribution. Map of counties and urban areas for preventable sight loss certifications in England (2017/19). Crude rates show per 100,000 population at risk compared with the benchmark for England: better—green, similar—yellow, worse—red, grey—not compared [4]. **a** All Sight Loss Certifications, **b** AMD, **c** Glaucoma, **d** Diabetic Eye Disease.

**Fig. 3:**
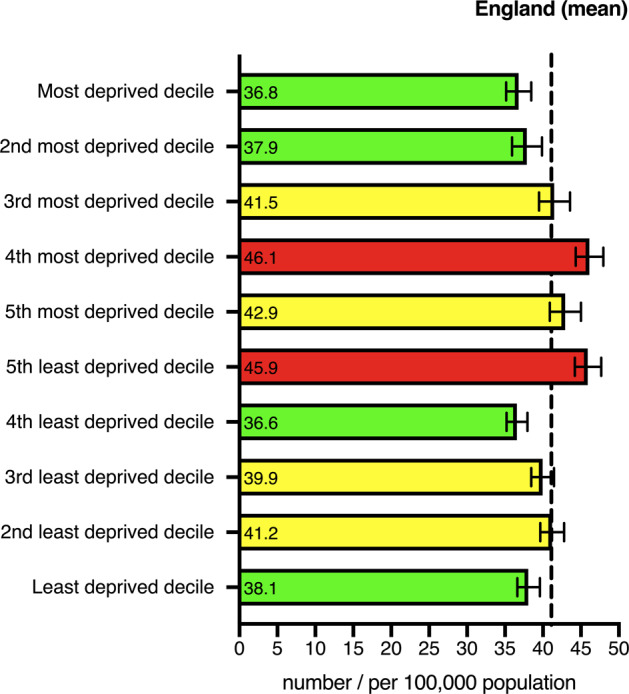
Preventable sight loss certifications in England (2017/18)—distribution by inequalities. Deprivation deciles by county and urban area, compared with the benchmark for England: better—green, similar—yellow, worse—red [4]. Deprivation deciles are defined using the Index of Multiple Deprivation 2015 local authority scores. They are created by ranking upper tier local authorities in England from most to least deprived and dividing these into ten categories with approximately equal numbers of local authorities in each.

The geographical variations in the rate of CVI certifications compared with the benchmark are illustrated in Figs. 2 and 3. This highlights potential inequalities in healthcare provision and the need to identify the causes of reversible sight loss in specific populations and use resources appropriately to address these causes. This is a complex issue which encompasses many variables including:

- Socioeconomic status
- Age of population
- Diet and lifestyle
- Ethnicity
- Education
- Access to opticians
- Access to hospital eye care services
- Efficient referral pathways between primary and secondary/tertiary care
- Capacity within the healthcare system to see, diagnose and treat patients promptly
- Eye Clinic Liaison Officer (ECLO) support
- Stringent CVI certification process

The number of vision impaired individuals that each regional Clinical Commissioning Group (CCG) has to support varies from 8 to 82 per 100,000 population. This demonstrates the need for CCGs to tailor their investments to their population’s needs. Directing local investment to the greatest health needs is essential to reducing inequalities and improving health outcomes. It is estimated that the cost of eye health and sight loss is approximately £28 billion per year [16]. This includes indirect costs such as lower employment rates, informal care and health issues associated with having sight loss [17].

### Wales

Certification of vision impairment became an NHS outcome measure for the Welsh Government in 2015. The aims of certification are to act as a mechanism of referral to the relevant local authority so that their name can be added to the register for assessment and support and provide epidemiological information about the causes of sight loss in Wales [18].

In 2017/18 there were 47 new vision impairment certifications per 100,000 Welsh residents. The number of new certifications with diabetic eye disease per 100,000 Welsh residents aged 12 or older has stayed the same since 2016/17, with there being three new CVIs per 100,000 Welsh residents aged 12 or older, during the 2017/18 financial year [19]. The incidence of new certifications per 100,000 population of persons with diabetes in Wales has almost halved between 2007 and 2015 [20].

**Fig. 4:**
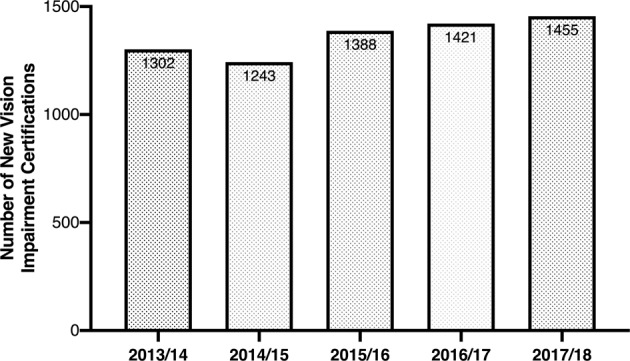
Number of new vision impairment certifications in Wales, 2013/14–2017/18 [21]. Data provided by the Welsh Government who hold copyright.

One hundred and twenty people per 100,000 aged 65 or over were certified with AMD and 14 people per 100,000 aged 40 or over with glaucoma [21] as the cause of their visual impairment. (Fig. 4)

### Role of the ECLO

In August 2017 the CVI was modified introducing a section specifying support from an ECLO. This was prompted by the Royal National Institute for the Blind’s campaign to have an ECLO in every eye clinic. ECLOs offer emotional and practical support to patients, family, friends and carers. They support patients throughout the certification process, ensuring that they understand their diagnosis and the support/welfare benefits they may be entitled to depending on their level of sight loss. They act as a bridge between the hospital and social care, ensuring patients know where to access support and feel more able to live an independent life with sight loss. ECLOs continue to support patients and their families after certification to ensure they receive social care needs assessments, mobility training and lighting support from rehabilitation officers.

### Access to data on CVI certifications and preventable sight loss indicators in England

The PHOF [4] provides essential data on health outcomes at both local and national levels. The data provided can be used to identify where populations are well served or where improvements are needed. This is invaluable information which can be used to improve services at a local level. It is important for ophthalmologists to be familiar with the preventable sight loss indicators, how to access this information and use it as a basis for improving outcomes locally.

The preventable sight loss indicators are classified under ‘Healthcare and Premature Mortality’ as E12a, E12b, E12c, E12d. When accessing the PHOF website, these indicators relevant for ophthalmologists can be swiftly viewed by selecting the ‘Healthcare and premature mortality’ domain and then scrolling down to indicators E12a, E12b, E12c, E12d. For each indicator once selected, a task bar at the top of the page allows the data trends, inequalities, population data and regional comparisons with be viewed for England. The annual indicator data and numbers of certifications can be downloaded as an Excel spreadsheet for county, unitary authority, district or region in England in 2010/11–2017/18.

### Looking to the future

The PHOF provides readily accessible data on recent trends in CVI certifications in the UK. Providers and commissioners of healthcare as well as individual ophthalmologists have a responsibility to be aware of this data and use it to minimise preventable sight loss in the populations they serve.

The recent trends have generally shown a decrease in the rate of CVI certifications, in particular with respect to AMD and diabetic retinopathy. However, the challenge remains as the number of people aged over 65 is predicted to increase by almost 50% over the next 20 years. By 2050 the number of people with sight loss in the UK is predicted to double, to over 4 million [16, 22]. This older age group is most at risk of sight loss.

Addressing current inequalities in care will help to combat the challenges faced. Easy access to eye care for all socioeconomic groups and all ethnic communities will help to identify patients at risk of preventable sight loss at an earlier stage. It has been found that certain ethnic minorities do not have the same level of access to these eye care services. Certain ethnic groups are at increased risk of sight loss from for example glaucoma. It is essential that these groups are educated and provided with accessible eye care. Lower income communities have a higher prevalence of sight loss which may partly be due to late presentation due to concerns with costs of travel and treatment [23–25].

Data on CVI trends are limited by the fact that it is only representative of the hospital population rather than the whole population. Ideally a national population-based survey of sight loss is required to provide the most accurate information on this. The certification process is being improved so that data collection is more reliable and uniform. Advances in the models of eye healthcare such as virtual clinics, telemedicine and more eye care in primary care will reduce the demand on hospital eye care services. EyesWise is a collaboration between the Elective Care Transformation Programme and Royal College of Ophthalmologists. It aims to ensure that patients who require consultant led care receive this as soon as possible, while those who do not need specialist care are managed appropriately without attending a specialised clinic, for example through virtual [26]. and community clinics [27]. However, patient education and early access to eye care remains a challenge to be overcome in particular for lower income communities and ethnic minorities.

Most recent data by NHS Digital has shown that ophthalmology is the largest outpatient specialty and now even exceeds orthopaedic surgery (NHS Digital). Ophthalmology provides almost 9 million outpatient appointments per year which is ~6% of all NHS outpatient appointments (RCOphth Workforce Census 2018). A ‘digital first’ approach to patient care and implementing strategies such as home monitoring and longer intervals for eye screening where appropriate will allow resources to be targeted to help those at highest risk of visual loss [28].

## Summary

### What was known before

- Baseline 2010/11–2011/12 sight loss indicator data were first published in August 2013 as part of the Public Health Outcomes Framework (PHOF) data release in England, and certification of vision impairment became an NHS outcome measure in Wales in 2015.

### What this study adds

- This study compares recent trends in new vision impairment certifications in 2017/18 against the baseline data from 2010/11 in England, and observes trends in new certifications in Wales. The trend in certifications for age-related macular degeneration (AMD) and diabetic eye disease has shown a decrease, whereas in glaucoma it has remained unchanged. The geographical variation in the rate of certifications highlights potential inequalities in healthcare and the need to specifically address the causes of reversible sight loss in certain regions.
